# What is the real predictor of coronary artery calcification in patients with chronic kidney disease?

**DOI:** 10.1080/0886022X.2022.2033266

**Published:** 2022-04-19

**Authors:** Yu Zhao, Wenyun Wang, Dong Zhilong

**Affiliations:** Department of Medicine, Northwest University for Nationalities, Lanzhou, PR China; Department of Pediatric Surgery, Second Hospital of Lanzhou University, Lanzhou, PR China; Department of Urology, Second Hospital of Lanzhou University, Lanzhou, PR China

Dear Editor,

We have read with great interest the paper entitled ‘Predictors of coronary artery calcification and its association with cardiovascular event in patients with chronic kidney disease’ by Wang et al. [1]. The authors conducted a prospective study to investigate the predictors of coronary artery calcification (CAC) (by using the Agatston score) and its association with cardiovascular events (CVE) in patients with stage 3–5 chronic kidney disease (CKD). In 131 patients, of which 35 were in the non-CAC group and 96 were in the CAC group, serum matrix Gla protein (MGP) was lower [10.50 (8.49, 13.53) ng/mL vs 18.88 (13.41, 23.23) ng/mL, *p* < .001] in the CAC group compared with the non-CAC group, respectively. Multivariate logistic regression analysis showed that lower serum MGP levels (OR 0.766, 95%CI 0.660–0.889, *p* < .001) were significantly associated with CAC. Multivariate Cox regression analysis demonstrated that higher MGP level was associated with a reduced risk for CVE (HR 0.340, 95%CI 0.124–0.933, *p* < .05). Kaplan-Meier analysis revealed that lower MGP level (≤11.69 ng/mL) was related to an increased risk for CVE (log-rank test, *p* < .001). We pay special attention to the MGP of this research because it caused us some confusion.

**Figure 1.**
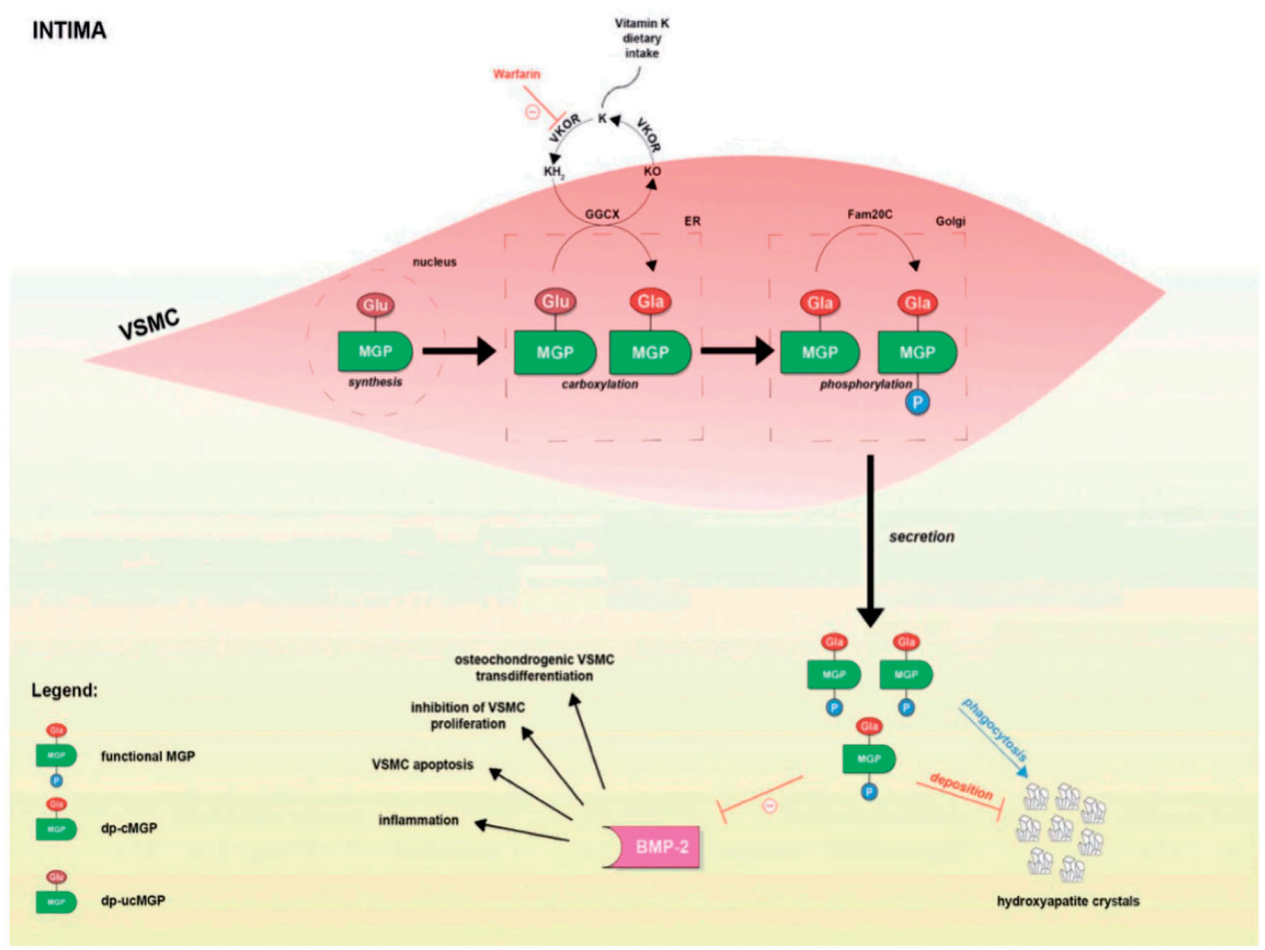
Post-translational modifications of MGP and mechanisms by which MGP is implicated in vascular calcification. Red lines depict inhibition of the process, whereas the blue line indicate stimulation (From Life (Basel). 2021, 11(8):737 [3]).

MGP, a small 12 kDa vitamin K-dependent protein, emerged as a potent vascular calcification inhibitor, which primarily synthesized and secreted by vascular smooth muscle cells. Two post-translational modifications of MGP are warranted in order to exert its functions. The first modification is a vitamin K-dependent γ-glutamate carboxylation, which enables binding of MGP to the crystal nuclei in hydroxyapatite and empowers MGP to binding and downregulation of bone morphogenetic protein-2 that will prevent VC [2]. After carboxylation, MGP need to undergo a Golgi-casein kinase phosphorylation to become biologically active. Phosphorylation is thought to be the most critical step in MGP activation, and plays a role in regulating MGP secretion into the extracellular environment. Only after γ-carboxylation and phosphorylation, MGP can gain the ability to calcium, hydroxyapatite and BMP-2 and thus inhibit vascular calcification (VC) (Figure 1) [3].

Depending on the carboxylation and/or phosphorylation status, MGP exists in four forms in circulation: phosphorylated-carboxylated MGP (p-cMGP), phosphorylated-uncarboxylated MGP (p-ucMGP), dephosphorylated-carboxylated MGP (dp-cMGP), and dp-ucMGP. Their respective affinity for calcium salts and accompanying calcification inhibitory activity may be quite different. However, it is confusing that the paper by Wang et al. did not differentiate between phosphorylated and dephosphorylated forms of ucMGP or cMGP.

Firstly, the fully active p-cMGP is known to be an inhibitor of VC, and the specific mechanism has been described previously. Dp-ucMGP represents a completely nonfunctional MGP with low affinity for calcium and stromal vesicles and thus free entry into the circulation. In previous studies, each 100-point increment in dp-ucMGP (in pmol/L) correlated roughly with a 10% increase in VC scores. A cross-sectional, prospective study in different stages of CKD including 107 patients (32% at CKD2-3, 31% at CKD4-5, 37% at HD) for follow-up median 846 days by Schurgers et al. [4]. and found that circulating dp-ucMGP was strongly associated with aortic calcification score (β0.000, 95%CI 0.000–0.001, *p* = .003), deterioration of renal function (*n* = 67, *r*^2^ = 0.268, *p* < .0001) and all-cause mortality (RR 1.05, 95%CI 1.00–1.09, *p* = .032).

Secondly, the partially inactive MGPs include both p-ucMGP and dp-cMGP. A review of literature found total ucMGP (t-ucMGP) which represents the p-ucMGP containing 1,2 or 3 phosphoserines as well as a range of degradation products. Schlieper et al. [5]. conducted a prospective cohort study consisted of 188 CKD patients and 98 healthy volunteers for mean follow-up 1104 days. Kaplan-Meier analysis showed that low dp-cMGP levels (<6139pmol/L) were associated with an increased all-cause and cardiovascular mortality risk (log-rank: *p* = .008 and *p* = .003, respectively). The multivariable Cox regression analysis showed that low plasma levels of dp-cMGP (<6139pmol/L) had HRs of 2.16 (95%CI 1.1–4.3, *p* = .027) for all-cause mortality and 2.74 (95%CI 1.2–6.2, *p* = .015) for cardiovascular mortality. Dalmeijer et al. [6]. conducted a cross-sectional study among 200 healthy women showed circulating dp-cMGP levels (β 0.70, 95%CI 0.20–1.20, *p* = .01 in crude analysis) and t-ucMGP levels (β −0.36, 95%CI −0.78 to 0.06, *p* = .09) were significantly associated with CAC in the entire study population.

Finally, we also found that the paper by Wang et al. [1]. did not consider the impact of renal replacement therapy on IL-6, did not test the level of vitamin K, did not mention whether CKD patients were taking vitamin K antagonists, such as warfarin, and did not conduct subgroup analysis of peritoneal dialysis patients and HD patients. Maybe the BioHybrid assay [7], a new cell-based high-content assay, to measure the vascular calcification propensity of an individual should be widely used.
